# High-risk follicular lymphomas harbour more somatic mutations including those in the AID-motif

**DOI:** 10.1038/s41598-017-14150-0

**Published:** 2017-10-25

**Authors:** Taku Tsukamoto, Masakazu Nakano, Ryuichi Sato, Hiroko Adachi, Miki Kiyota, Eri Kawata, Nobuhiko Uoshima, Satoru Yasukawa, Yoshiaki Chinen, Shinsuke Mizutani, Yuji Shimura, Tsutomu Kobayashi, Shigeo Horiike, Akio Yanagisawa, Masafumi Taniwaki, Kei Tashiro, Junya Kuroda

**Affiliations:** 1 Division of Hematology and Oncology, Department of Medicine, Kyoto Prefectural University of Medicine, Kyoto, Japan; 2 Department of Genomic Medical Sciences, Kyoto Prefectural University of Medicine, Kyoto, Japan; 3 Department of Hematology, Matsushita Memorial Hospital, Osaka, Japan; 4 Department of Hematology, Japanese Red Cross Kyoto Daini Hospital, Kyoto, Japan; 5 Department of Surgical Pathology, Kyoto Prefectural University of Medicine, Kyoto, Japan

## Abstract

We investigated clinical and genetic characteristics of high-risk follicular lymphoma (FL), that lacked evidence of large cell transformation at diagnosis, in the rituximab era. First, we retrospectively analysed the clinical features of 100 patients with non-transformed FL that were consecutively treated with rituximab-containing therapies in a discovery cohort. The presence of either peripheral blood and/or bone involvement was associated with short progression-free survival. This was confirmed in a validation cohort of 66 FL patients. Then, whole exome sequencing was performed on randomly selected 5 high- and 9 standard-risk FL tumours. The most common mutational signature was a CG > TG substitution-enriched signature associated with spontaneous deamination of 5-methylcytosine at CpG, but mutations in WA and WRC(Y) motifs (so-called activation-induced cytidine deaminase (AID) motifs) were also enriched throughout the whole exome. We found clustered mutations in target sequences of AID in the *IG* and *BCL2* loci. Importantly, high-risk FLs harboured more somatic mutations (mean 190 vs. 138, *P* = 0.04), including mutations in WA (33 vs. 22, *P* = 0.038), WRC (34 vs. 22, *P* = 0.016) and WRCY motifs (17 vs. 11, *P* = 0.004). These results suggest that genomic instability that allows for emergence of distinct mutations through AID activity underlies development of the high-risk FL phenotype.

## Introduction

Non-Hodgkin lymphoma (NHL) is the most prevalent hematologic malignancy, and follicular lymphoma (FL) is the most common subtype of indolent NHLs, accounting for approximately 20% of all NHLs. During the last decade, the advent of immunochemotherapy incorporating anti-CD20 monoclonal antibody, such as rituximab (Rit), has drastically changed the treatment strategy for FL, and resulted in a marked improvement in overall prognosis^1^. Indeed, first-line immunochemotherapy followed by Rit maintenance therapy, the current standard of care for FL, has significantly prolonged progression-free survival (PFS) and is also expected to account for the improvement in overall survival (OS)^2–4^. However, FL still remains mostly incurable, and many patients experience recurrences that require repeated salvage therapies during a lifelong disease course. Although a series of novel agents has improved the outcomes of relapsed patients, FL with a short PFS after the initial therapy requires more intensive and more types of salvage therapies, including autologous and allogeneic hematopoietic stem cell transplantation. Those secondary salvage therapies can considerably affect the patients’ physical, mental, social, and economic status. One of the leading and well-defined causatives for an aggressive disease phenotype with poor treatment outcome in FL is histologic large cell transformation which is sometimes already present at diagnosis, or emerges at approximately 2–3% per year during the disease course^5,6^. Transformed FLs can be relatively easily diagnosed by a histological assessment of biopsied specimens and by its aggressive clinical manifestations, and should be treated as high-grade NHLs, like diffuse large B-cell lymphoma (DLBCL)^5,6^. In contrast to the transformed variant FL, the serious issue facing non-transformed FL has been that a minor, but specific population of patients suffers from an extremely short PFS (within 1 to 2 years) due to either their primary resistance to immunochemotherapy or to early disease relapse despite the transient disease control. Indeed, a large retrospective study demonstrated that FL patients with early progression after R–CHOP showed significantly poorer OS than those without early progression^7^. Conventional prognostic indexes, such as the Follicular Lymphoma International Prognostic Index (FLIPI) for OS prediction or the modified FLIPI2 for OS and PFS prediction, have been widely used for FL patients in daily practice^8,9^. However, clinical characteristics that are not included in FLIPI might also have a prognostic impact in the Rit era^8,9^.

The t(14;18)(q32;q21) and the resultant *BCL2/IGH* fusion gene are hallmarks of 80–90% of FLs, and play a fundamental role in FL pathogenesis through constitutive BCL2 overexpression. In addition to deregulated BCL2 overexpression, recent studies have revealed the presence of various additional mutations associated with epigenetics (*CREBBP*, *EZH2 KMT2D*, *MEF2B*, *ARID1A*, etc.), JAK/STAT signalling (*SOCS1*, *STAT6*, etc.), BCR/NF-κB signalling (*CARD11*, *TNFAIP3*, etc.), and the immune response (*TNFRSF14*) in FL tumours at diagnosis and were suggested to be involved in both development and disease progression^10–12^. Although several studies have attempted to clarify the molecular mechanisms underlying histologic transformation in FL^13^, the genetic/molecular features of high-risk non-transformed FL with a short PFS have not been clarified. To further improve the treatment outcome of FL patients, it is essential to identify the clinical features and the molecular basis for development of the high-risk disease phenotype in FL, excluding transformed FL. To this end, we first tried to identify the high-risk disease phenotype in FL, excluding FL cases showing evidence of transformation at diagnosis. Based on the findings, we next investigated the genetic features of the high-risk FLs defined by our criteria using whole exome sequencing (WES).

## Results

### Patients, treatment outcome, and generation of a prognostic model for PFS in FL

The patients’ information of the discovery cohort is summarized in Supplementary Table S1. In brief, the median age of 100 patients was 61 years old, and a t(14;18) translocation was positive in 65 of 74 evaluable patients (88%). Sixty-six patients (66%) were classified as being in the advanced stage according to the Ann Arbor Staging system, and 47 (47%) had extranodal involvements, including 31 with bone marrow (BM) involvement, at diagnosis. Twenty-nine of 97 (30%) and 11 of 51 (22%) evaluable patients were classified as high-risk according to both FLIPI and FLIPI2, respectively.

**Figure 1:**
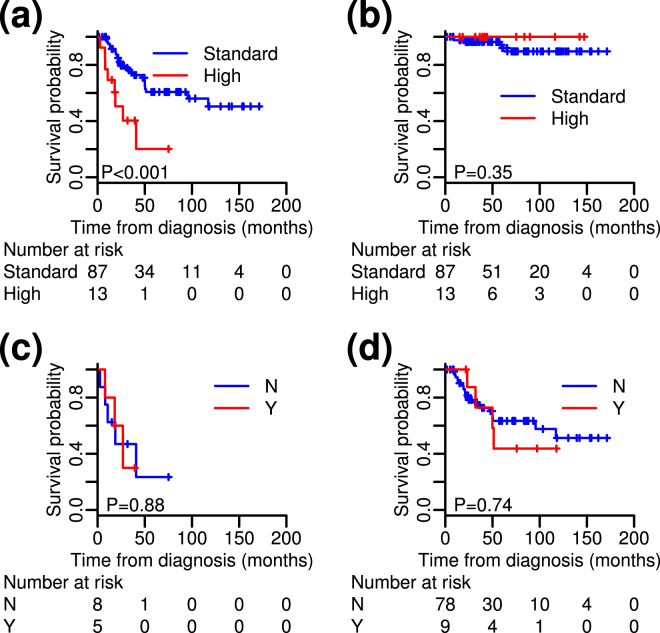
Progression-free survival (PFS) and overall survival (OS) of patients with non-transformed follicular lymphomas (FLs). **(a**) PFS and (**b**) OS of high-risk and standard-risk FLs in the discovery cohort. (**c,d**) Comparisons of PFS between high- (**c**) and standard (**d**) -risk patients who were (Y) and were not (N) subjected to whole exome sequencing (WES). PFS did not differ significantly between the Y and N groups in high- and standard-risk FLs.

With the median follow-up period of 61 months, 84 (84%) of the patients received either Rit-containing immunochemotherapy or Rit monotherapy as the first-line treatment, while 16 (16%) of the patients were initially subjected to watchful waiting (Supplementary Table S2). In 80 evaluable patients, the overall response rate to the first-line therapy was 96%, including 76% with a complete response, while 3 remained stable. The 5-year OS and PFS of the entire cohort were 94.9% and 56.1%, respectively (Supplementary Fig. S1a,b). One patient died of FL progression, while other causes of deaths were not associated with FLs. Only 1 patient subsequently developed large cell transformation at relapse. To identify FL patients who are at high-risk for shorter PFS, the prognostic significance of sites of extranodal involvement was evaluated. Univariate analysis revealed that the presence of at least one of three extranodal involvements, i.e., peripheral blood (PB) (N = 7), bone (N = 6), or lung (N = 1), significantly associated with poor PFS (Supplementary Table S3). In multivariate analysis, we evaluated the significance of PB and bone involvements as the variables adjusted for high risk of FLIPI, while lung was excluded from the analysis because only 1 patient had lung involvement. PB and/or bone involvements were found to significantly associate with short PFS (Supplementary Table S3 and Fig. 1a). Indeed, 13 high-risk patients with PB and/or bone involvements at diagnosis showed a significantly shorter median PFS of 27.2 months compared with that of the 87 standard-risk patients (who have not yet reached the median), despite no significant diversity in terms of the first-line treatment. In contrast, all of the 13 high-risk patients were rescued by a series of salvage therapies during their observation periods, and OS was not significantly different between the 13 high-risk and 87 standard-risk patients (Fig. 1b). The clinical characteristics of the high- and standard-risk patients defined in this study are summarized in Supplementary Table S4. Although histologic grade or the presence of bulky mass were not significantly different between both risk groups, the high-risk patients according to our criteria had more nodal involvement sites and were assigned a higher risk based on the FLIPI; 5 of the 13 were intermediate and 7 of the 13 were high risk.

To confirm the prognostic significance of the PB and bone involvements, we made the validation cohort consisted with 66 patients who treated in Japanese Red Cross Kyoto Daini Hospital. Comparing the discovery and validation cohorts, there were no significantly differences in their clinical characteristics except histological grade: More patients represented grade 3a in the validation cohort (41%) than in the discovery cohort (18%) (*P* < 0.001). The 5-year OS and PFS of the entire validation cohort were 94.3% and 53.0%, respectively, representing no significant differences between two cohorts (*P* = 0.45 for OS and *P* = 0.32 for PFS) (Supplementary Fig. S1a,b). Overall, in the validation cohort, 11 (17%) and 55 (83%) patients were classified as the high- and standard-risk, respectively. We confirmed that the high-risk patients of the validation cohort were significantly associated with inferior median PFS of 57.7 months compared with that of the standard-risk patients of 71.0 months (*P* = 0.04).

### High-risk FLs had more somatic mutations compared with that of the standard-risk FLs

As an initial exploratory study, we next investigated the genetic characteristics of the high-risk FLs in comparison with the standard-risk FLs. We randomly selected 5 high-risk patients (H1-H5) and 9 standard-risk patients (S1-S9) for WES analysis out of the total 100 patients in the discovery cohort. The backgrounds and clinical characteristics of the 14 patients for WES are summarized in Supplementary Table S5. PFS of patients subjected to WES were not significantly different from the rest patients both in the high- and standard-risk groups (Fig. 1c,d).

**Figure 2:**
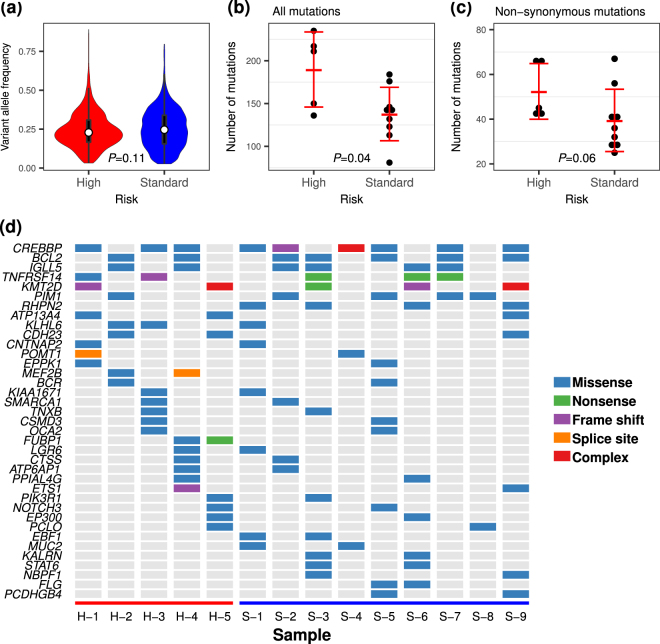
Overview of the results from whole exome sequencing. A violin plot (**a**) showed no difference in median variant allele frequency between the risk groups, whereas the numbers of all somatic mutations (**b**) were significantly higher in high-risk FLs than in standard-risk FLs and non-synonymous mutations (**c**) tended to be increased in high-risk FLs. (**d**) Recurrent mutated genes are listed in the tile plots and mutation types are indicated by the coloured tile.

In WES, we achieved an average on-target rate of 80.8% (range; 75.2–85.3), an average depth of coverage of x291.2 (range; 237.1–380.4) and an average coverage rate over x10 of 98.6% (range; 98.0–99.1) for tumour samples, an on-target rate of 80.8% (range; 76.2–86.7), an average depth of coverage of x99.2 (range; 80.8–115.2) and an average coverage rate over x10 of 91.4% (range; 86.4–92.9) for germline samples (Supplementary Table S6). The alternative allele frequencies of all variants, which were supposed to represent the constituent rate of each clone, from the tumour samples were not significantly different between both groups (*P* = 0.11, t-test) (Fig. 2a). When comparing the number of genes possessing somatic mutations between both groups, the high-risk FLs had more somatic mutations than the standard-risk FLs. The mean numbers of total mutations in the high-risk and in the standard-risk groups were 190 and 138 (*P* = 0.04) (Fig. 2b), respectively, and the mean numbers of non-synonymous mutations were 52 and 39 (*P* = 0.06), respectively (Fig. 2c and Supplementary Fig. S2a,b).

### Functional assessment of mutated genes in high- and standard-risk FLs

Recurrent non-synonymous variants, including missense mutations, nonsense mutations, frame-shift mutations, and aberrant splice regions (listed in Fig. 2d and Supplementary Fig. S2a,b), were further assessed for their functional significances. A number of recurrently mutated genes identified from 14 patients in this study were found to agree with the results reported in previous studies for FL^10–12,14^. MutSigCV found that mutations in *CREBBP* (false discovery rate (FDR); 0.015) and *TNFRSF14* (FDR < 0.001) genes were driver mutations among recurrently mutated genes. Although activation-induced cytidine deaminase (AID)-targeted genes, such as *BCL2*, *PIM1*, and *IGLL5*, were frequently mutated, those mutations were found to be less potent as driver mutations and were not predicted as protein damaging mutations according to the variant effect *in silico* tools (Fig. 2d and Supplementary Table S7)^15,16^. Interestingly, *MEF2B* and *FUBP1* were identified as recurrently damaging mutated genes only in the high-risk FLs. In addition, the hypergeometric test on all of the non-synonymous mutated genes from 14 FLs resulted in 71 significantly enriched gene ontologies (GOs), as represented by the terms “cell development and differentiation”, “regulation of cell death”, “programmed cell death and apoptosis”, “signalling pathways”, “immunity mediated by lymphocytes, including B cells”, “hematopoiesis”, and “differentiation of leukocytes, including lymphocytes” (Supplementary Table S8). In contrast, pathway analysis demonstrated that mutated genes were enriched in the Notch and BCR signalling pathways (Supplementary Table S9). However, we were not able to identify GOs or signalling pathways specific to the high-risk FL patients that could explain the different outcomes of the disease.

### Common mutational signatures and mutations in target sequences of AID

**Figure 3:**
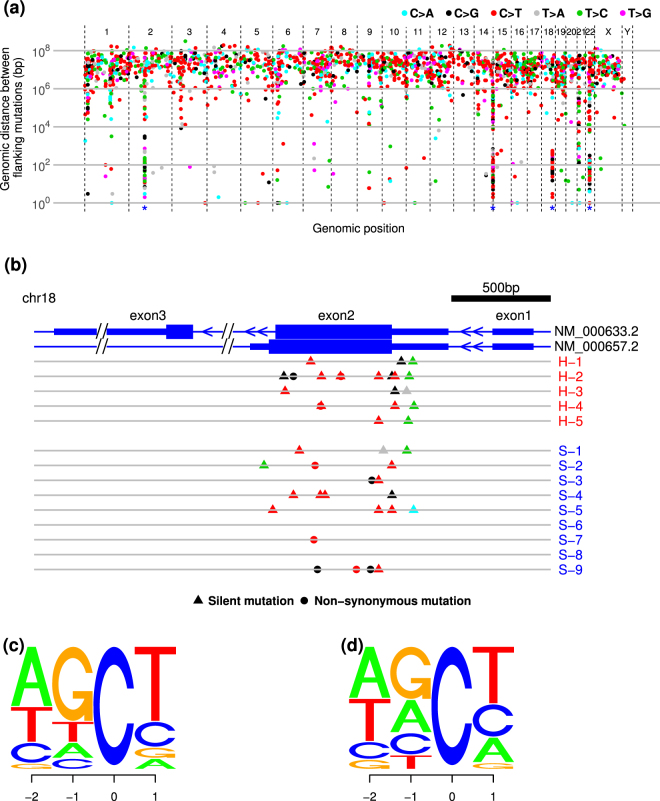
Clustering somatic mutations. (**a**) Rainfall plots of single nucleotide variants (SNVs) for 14 FLs. Each dot represents a single SNV coloured by the type of substitutions and ordered by the human genomic position on the horizontal axis. The vertical axis represents the genomic distance between flanking mutations of individual samples on a log scale. (**b**) Clustering mutations in the *BCL2* locus. Each dot indicates a non-synonymous mutation and each triangle indicates a silent mutation, coloured by substitution pattern as in **a**. Wide blue bands of each transcript represent coding sequences and narrow bands represent 5′ and 3′ untranslated regions (UTRs). (**c**,**d**) C > T mutations in *BCL2* (**c**) and *IG* (**d**) loci. The size of each letter represents the frequency of base constitutions. The -2 and -1 indicate positions 5′ from C > T mutations and +1 indicates the 3′ position.

By tracing the rainfall plot, we confirmed the presence of clustered mutations on chromosome 2p12 (involving the *IGK* locus), 14q32 (*IGH*), 18q21 (*BCL2*), and 22q11 (*IGLL*) in both the high- and standard-risk groups (Fig. 3a). Given that clustered mutations in the *IG* locus were considered to be the results of somatic hypermutation, we next focused on mutations in the *BCL2* locus. As shown in Fig. 3b, somatic mutations were clearly clustered on exon 2 and most substitutions were C > T or T > C transitions and C > G transversions. Moreover, the common sequence of C > T mutations corresponded to the so-called AID motif: WRC or WRCY, where W = A/T, R = A/G, and Y = C/T (Fig. 3c)^17^. In addition, C > T transitions in the *IG* locus were also enriched in the AID motif (Fig. 3d).

**Figure 4:**
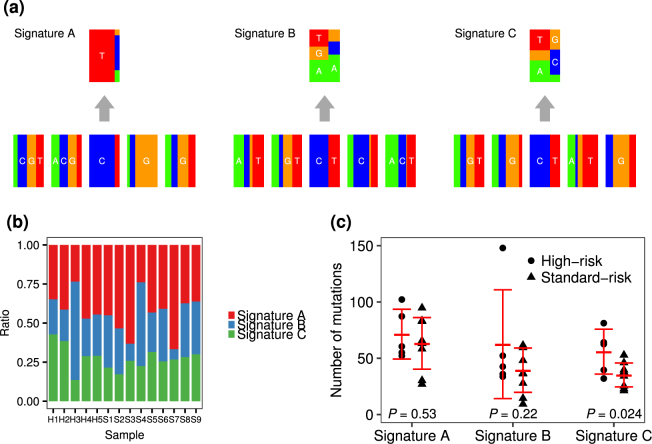
Mutational signatures of 14 FLs. (**a**) Three simplified mutational signatures extracted from 14 FLs based on sequences of the −2, −1, +1 and +2 positions and six substitution patterns, defined as Signatures A, B and C. The compositions of each signature in an individual tumour did not differ significantly between high- and standard-risk FLs (**b**), while the numbers of mutations of Signature C were significantly higher in high-risk FLs (**c**).

To investigate the possible involvement of additional overlapping mutational processes other than AID-induced mutation, the mutational signatures were analysed. In our samples, the common mutational signature according to the catalogue of somatic mutations in cancer (COSMIC) (http://cancer.sanger.ac.uk/cosmic/signatures) was signature 1, which is the result of an endogenous mutational process initiated by spontaneous deamination of 5-methylcytosine at CpG, while other mutations were classified into various signatures, including those correlated with DNA mismatch repair (signatures 6, 15 and 26), transcription-coupled nucleotide excision repair (signature 7) or associated with AID activity (signature 9) (Supplementary Fig. S3). Next, we selected three common mutational signatures from 5-bp contextual sequences, using a simplified and parameter-reduced method to maintain statistical stability. A CG > TG substitution-enriched signature (designated as “Signature A”) was the most common form in the 14 FL tumours, and the third most common form (designated as “Signature C”) was similar to signature 9 categorized by COSMIC; these signatures were considered to be associated with AID and error-prone polymerase η activities characterized by a T > C/G transition in the NpTpA/T context (Fig. 4a and Supplementary Fig. S4a,b). The composition of the three signatures in individuals did not differ significantly between high- and standard-risk FLs, but the mean number of mutations of Signature C was significantly higher in high-risk FL (56 vs. 35, *P* = 0.024) (Fig. 4b,c).

**Figure 5:**
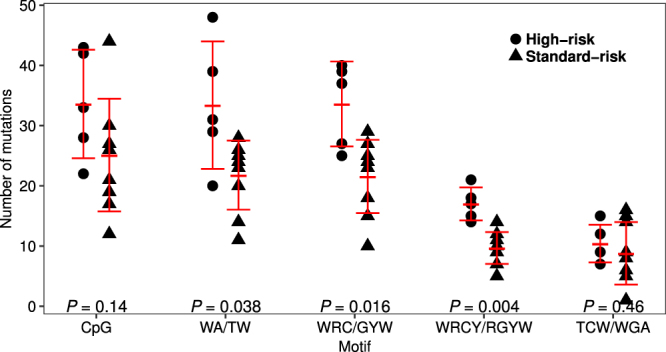
Mutational load in known motifs. Dot plots of the numbers of somatic mutations in CpG, WA, WRC, WRCY and TCW motifs. Dots indicate high-risk and triangles indicate standard-risk. Mutations in the *IG* loci are excluded.

Next, we investigated the mutation patterns by taking contextual sequence motifs into account. Mutations of CpG, WRC(Y), and WA motifs were significantly enriched throughout the whole exome, but mutations of CpG and WA motifs were less frequent in the *IG* or *BCL2* locus compared with other loci (Table 1). The mutational rates in WRC(Y) motifs in the *IG* and *BCL2* loci were higher than those calculated in the whole exome. In contrast, the mutation rate of SYC motifs, i.e. AID cold spots, was significantly lower only in the *IG* locus. The mutation rate in the TCW motif, i.e. APOBEC target, was relatively low throughout the whole exome, including the *IG* and *BCL2* loci. We next analysed the impact of each mutational process. The mean numbers of mutations excluding the *IG* loci in the WA (33 vs. 22, *P* = 0.038), WRC (34 vs. 22, *P* = 0.016) and more refined WRCY (17 vs. 10, *P* = 0.004) motifs were significantly increased in high-risk cases, whereas there was no difference in those in CpG (34 vs. 25, *P* = 0.14) and TCW (10 vs. 9, *P* = 0.46) motifs between the high- and standard-risk cases (Fig. 5). To ascertain whether these findings are depend on t(14;18)(q32;q21), we re-compared the number of mutations between the high- and standard-risk FLs excluding one high-risk FL and one standard-risk FL those who were not assessed chromosomal abnormality or t(14;18)(q32;q21)-negative. As the results shown in supplementary Fig. S5, the total number of mutations and mutations in the AID-motif were increased in the high-risk FLs.

**Table 1:** Comparison of the mutational frequencies with expected frequencies of motif sequences in whole exon, immunoglobulin and *BCL2* loci.

motif	Whole exon			*IG*			*BCL2*		
	N (probability)	Expected freq.	*P*-value	N (probability)	Expected freq.	*P*-value	N (probability)	Expected freq.	*P*-value
CpG	445 (0.203)	0.087	3.19E-63	10 (0.035)	0.056	7.58E-04	2 (0.048)	0.055	1.00E + 00
WA/TW	422 (0.193)	0.110	6.73E-30	59 (0.209)	0.141	1.98E-03	6 (0.143)	0.150	1.00E + 00
WRC/GYW	462 (0.211)	0.115	1.94E-37	100 (0.355)	0.108	3.99E-28	17 (0.405)	0.108	4.67E-26
WRCY/RGYW	246 (0.112)	0.044	7.79E-40	74 (0.262)	0.035	3.11E-42	14 (0.333)	0.036	1.46E-10
TCW/WGA	141 (0.064)	0.080	7.27E-03	10 (0.035)	0.085	1.75E-03	5 (0.119)	0.085	4.00E-01
SYC/GRS	289 (0.132)	0.141	2.55E-01	13 (0.046)	0.119	4.35E-05	1 (0.024)	0.110	8.27E-02

*IG*, immunoglobulin; freq., frequency.

## Discussion

In this study, we identified prognostic factors for FL in the Rit era and investigated the genetic features and oncogenic processes of FLs, especially those associated with disease aggressiveness of non-transformed FLs. The prognostic factors found in this study is distinct in several ways from the conventional models for FL, such as FLIPI, or FLIPI2. Firstly, our model was developed using only non-transformed FL patients who were subjected to first-line immunochemotherapy containing Rit or to watch and wait according to the GELF criteria. Secondly, our study disclosed the prognostic impact of specific extranodal involvements, bone and/or PB at diagnosis, those were in accordance with the findings in previous studies^18–20^. The third distinctive aspect of this study was that, while FLIPI was developed for prediction of OS, our model was developed for the purpose of predicting PFS by evaluating the predictive value of each variable for PFS. Indeed, over 40% of the high-risk patients according to our criteria were classified as low or intermediate risk in FLIPI in both the discovery and validation cohorts (Supplementary Table S4). Despite the diversity of PFS, OS did not differ significantly between high- and standard-risk FL in our cohorts using our model, indicating that high-risk patients were successfully rescued by various salvage strategies. Indeed, no high-risk patient died of FL during the observation period, again illustrating the improvement of OS in the current daily practice with increasing treatment options for FL. Even though our clinical analysis in discovery and validation cohorts has potential limitations including small sample sizes, being retrospective and inherent biases such as lower frequency of BM involvements and higher proportion of grade 3a in the validation cohort, the proposed model nonetheless allowed further assessment of genetic features of the high-risk FLs cases defined by our criteria.

Recent studies using next generation sequencing analysis have suggested that the mutations in epigenetic regulators, such as *CREBBP* and *EZH2*, were associated with early disease development as driver mutations^10,11,21^. Furthermore, the mutations in several key functional genes involved in BCR to NF-κB signalling, B cell development, JAK-STAT signalling, and immune response, have also been suggested to play crucial roles in the pathogenesis of FL^12,22^. Several studies have identified various genetic abnormalities affecting the prognosis and transformation of FL^12,23,24^. Moreover, the combination of clinical characteristics such as FLIPI or progression in 24 months and genetic abnormalities has been described to predict the outcome of FL more precisely^23,25^. However, the landscape of clinical and genetic features associated with poor outcome of FL has not revealed sufficiently. Although our study is limited in terms of patient numbers and by the use of WES rather than whole genome sequencing, our results suggest a number of interesting differences between high-risk and standard risk FLs defined by our clinical criteria. In line with the past studies, we found that FL tumour cells had a number of mutations in addition to *BCL2/IGH* rearrangement, and that recurrent variants were enriched in genes associated with epigenetic regulation, BCR signalling, B cell development, immune responses, and programmed cell death, and defined *CREBBP* and *TNFRSF14* as driver mutations in our overall cohort.

Mutational signatures have been proposed to define mutational processes in various cancers. We found that mutational signatures responsible for spontaneous deamination of 5-methylcytosine at CpG and AID activity were the general forms and the high-risk FLs had been more strongly influenced by AID under tumour development. Our result suggested that each mutational process might have different impact on the prognosis and clinical characteristics.

AID, a member of AID/APOBEC family of deaminases, is essential for the somatic hypermutation (SHM) and class-switch recombination (CSR) of IG genes^26^. Although the mechanism by which AID triggers SHM and CSR has been explained by two distinct models: the DNA deamination model and RNA editing^27^, AID has been proposed to cause mutagenic U:G mismatches in DNA and to mediate off-target mutations outside IG genes in B cell malignancies^28,29^. Although the BER and MMR pathways faithfully repair U-G mismatches, replication of a U-G mismatch leads to a C > T transition. Furthermore, error-prone polymerase η induces accumulation of mutations during BER and MMR. Numerous *in vitro* and *in vivo* studies have revealed hot spots responsible for AID-induced mutations, i.e. WA/TW and WRCY/RGYW (or WRCH/DGYW)^17,30,31^. In FL, AID plays crucial roles in disease development and progression^32–34^, and more mutations have been identified in motifs recognized by AID and by APOBEC at relapse than that at diagnosis^10^. In addition, FL patients with *BCL2* mutations accompanied by high AID expression have a poor prognosis^35^, and patients with accumulated mutations in AID targeted genes are at high risk for transformation. Although recent studies have referred to AID-induced mutations and prognosis of FLs, this is the first study to identify the relationship between prognosis and AID-induced genomic instability throughout the whole exome and involving both well-known AID-targeted genes and other genes with potential AID-targeted motifs, supported by evidence from motif enrichment analyses comparing the whole exome and clearly AID-targeted gene loci. Furthermore, we found that AID-induced genomic instability was associated with a poor prognosis independently of transformation. A recent study demonstrated FL tumours harbour excess mutations in AID-motif overlapping the CpG methylation sites^36^. In line with this study, our results from mutational signature analysis and motif analysis revealed increased number of mutations in AID-motifs and CpG, while our finding gave insights into the role of AID on the aggressiveness of FLs. Collectively, AID activity should be one of the pivotal components determining the biological features of extranodal localization and clinical outcomes in FLs. Although we could not validate our results using public datasets because those datasets lack the detailed clinical information like PB or bone involvement at diagnosis, further validation using next generation sequencing is desired. It would be also interesting to investigate whether AID activity could serve as a surrogate marker for genetic instability that may affect the clinical outcome of FLs in the future.

As the number of genes possessing somatic mutations was more frequent in the high-risk patients rather than in the low-risk patients, there were some interesting genes specific to the high-risk patients, such as *MEF2B* and *FUBP1*, although these mutated genes were not commonly shared even within the high-risk patients. The *MEF2B* mutation deregulates the expression of *BCL6* oncogene and contributes to lymphomagenesis in DLBCL. Considering the recurrent *MEF2B* mutations in DLBCL^37^, it is conceivable that mutated *MEF2B* also contributes to disease aggressiveness in FL. Additionally, recent study revealed frequent *MEF2B* mutation in transformed FLs. The *FUBP1* gene is an important co-transcriptional factor of the *MYC* oncogene^38^, and, therefore, mutated *FUBP1* may accelerate FL. Importantly, the *MEF2B* and *FUBP1* mutations did not represent the AID signature (or APOBEC signature). Thus, our results propose that the high-risk FLs had more mutations than the standard-risk FLs due to multifaceted stimuli consisting of the increased mutations mediated by AID. These findings imply the presence of an endogenous and/or exogenous error-prone environment that may eventually cause the accumulation of more secondary driver/oncogenic mutations determining the fate of the high-risk FLs.

In conclusion, our results highlighted several aspects of genetic features which may explain the biological basis for the development of the high-risk phenotype in FL as the follows: (i) high- and standard-risk FLs share mutations associated with similar biologic processes, such as epigenetics and BCR-NFκB signalling, (ii) both risks share similar, or, perhaps a common AID mutational signature, suggesting a common etiology between two cohorts, however, (iii) the high-risk FLs had more somatic mutations compared to that of the standard-risk FLs in AID-targeted as well as in non-AID-targeted regions, and (iv) more variants in high-risk disease include random, but meaningful mutations for developing an aggressive phenotype in lymphoid malignancies. These findings suggested that the high-risk FLs might have more harmful genetic instabilities.

## Methods

This study was conducted in accordance with the ethical principles of the Declaration of Helsinki, and was approved by the Ethical Review Board of Kyoto Prefectural University of Medicine and the Ethical Review Board of Japanese Red Cross Kyoto Daini Hospital. All specimens from tumours and peripheral blood mononuclear cells (PBMCs) were obtained with informed consent from the patients.

### Patients and clinical assessment

In the discovery cohort, we retrospectively analysed 100 patients who were newly diagnosed by independent pathologists as FL by histologic assessments according to the WHO classification^39^ and treated in Kyoto Prefectural University of Medicine between April 2001 and March 2014. The following patients were excluded from this investigation: those with histologic grade 3b, those who showed the histologic component of large cell lymphoma at diagnosis regardless of later development to large cell transformation, those who were treated with first-line chemotherapy in other institutes, and those who were treated with first-line chemotherapy without Rit. The validation cohort included 66 patients who were consecutively diagnosed and treated in Japanese Red Cross Kyoto Daini Hospital between September 2004 and August 2015. The exclusion criteria of the validation cohort were the same with those adapted in the discovery cohort. The detailed diagnostic procedures are shown in the Supplementary Methods.

### Statistical analyses

PFS was defined as either the time from diagnosis to documented disease progression or the date of death from any cause, whichever occurred first. OS was calculated from diagnosis to death from any cause. OS and PFS were estimated using the Kaplan-Meier method. Log-rank tests were performed to evaluate the prognostic impact of each variable by univariate analysis. The multivariate Cox proportional-hazards regression model was used to estimate hazard ratios for evaluating the impact of extranodal involvements on PFS. All the variables for the patient cohorts were compared across the groups using Fisher’s exact test for categorical variables and the Student’s t-test. All statistical calculations were performed using R (version 3.2.3).

### Sample collection, library preparation and analysis of WES data

Genomic DNA was extracted from cryopreserved tumour specimens at diagnosis and from matched normal PBMCs at complete remission, followed by WES library preparation using SureSelectXT Target Enrichment System Kit (Agilent Technologies, Santa Clara, CA, USA) and SureSelectXT Human All Exon v5 Capture Library Kit (Agilent Technologies) for Illumina Multiplexed Sequencing. The sequencing was performed with the HiScanSQ (Illumina, San Diego, CA, USA) using the 100-bp paired-end method. Variant detection was performed by three programs with different algorithms: MuTect (version 1.1.4), VarScan2 (version 2.3.8), and Strelka (version 1.0.14)^40–42^. Among the candidate variants extracted by those algorithms, we considered a true variant when at least two of the three programs defined it as a positive variant. To determine common mutational signatures, we used an R package pmsignature (version 0.2.1) with default parameters^43^. To define enriched mutational motifs in the whole exome, immunoglobulin locus, and *BCL2* locus, a Fisher exact test was used to compare the expected frequency of each motif with the observed mutation rate. A Wilcoxon rank sum test was used to compare numbers of mutations between the groups. A *P*-value of less than 0.05 was considered significant. See the Supplementary Methods for additional details of next generation sequencing library preparation, sequencing and analysing data.

## Electronic supplementary material

Suppelementary Information

Table S7
