# Supplementary material for: High-risk follicular lymphomas harbour more somatic mutations including those in the AID-motif
Source: Sci Rep. 2017 Oct 25;7:14039. doi: 10.1038/s41598-017-14150-0 (PMC5656578; doi:10.1038/s41598-017-14150-0)
Supplement: Supplementary file 1 — Suppelementary Information [file 41598_2017_14150_MOESM1_ESM.pdf]

High-risk follicular lymphomas harbour more somatic mutations including those in the AID-motif

Taku Tsukamoto[1,*], Masakazu Nakano[2,*], Ryuichi Sato[2], Hiroko Adachi[2], Miki Kiyota[1, 3], Eri Kawata[4], Nobuhiko Uoshima[4], Satoru Yasukawa[5], Yoshiaki Chinen[1], Shinsuke Mizutani[1], Yuji Shimura[1], Tsutomu Kobayashi[1], Shigeo Horiike[1], Akio Yanagisawa[5], Masafumi Taniwaki[1], Kei Tashiro[2], Junya Kuroda[1]

1. Division of Hematology and Oncology, Department of Medicine, Kyoto Prefectural University of Medicine, Kyoto, Japan

2. Department of Genomic Medical Sciences, Kyoto Prefectural University of Medicine, Kyoto, Japan

3. Department of Hematology, Matsushita Memorial Hospital, Osaka, Japan

4. Department of Hematology, Japanese Red Cross Kyoto Daini Hospital, Kyoto, Japan

5. Department of Surgical Pathology, Kyoto Prefectural University of Medicine, Kyoto, Japan

* These authors contributed equally to this work.

Supplementary methods

Patients and diagnostic procedures

We retrospectively analyzed 103 patients in the discovery cohort and 68 patients in the validation cohort. The Groupe pour l'Etude de Lymphome Folliculaire (GELF) criteria were basically utilized for the determination of whether to initiate systemic immunochemotherapy [1]. Extranodal involvements, except bone marrow (BM) and peripheral blood (PB), were examined by a computed tomography (CT) scan and/or 2-deoxy-2-[fluorine-18]fluoro-D-glucose integrated with CT (^{18}F-FDG PET/CT) based on the recommendations of the 11th International Conference on Malignant Lymphoma [2]. Involvements of the BM and PB were assessed cytologically by counting 500 and 100 cells under a light microscope, respectively, and BM was also assessed by histologic examination. The criteria of the conventional prognostic indexes, i.e., Follicular Lymphoma International Prognostic Index (FLIPI) and follicular lymphoma international prognostic index-2 (FLIPI2), and the definition of treatment response and disease progression were described previously [3-6]. The patients' clinical data were obtained from their medical records.

Chromosomal analysis

Giemsa-banding was performed on metaphase spreads of biopsied specimens, and the karyotype was described in accordance with the International System for Human Cytogenetic Nomenclature (ISCN) (2013) [7]. Double color fluorescence *in situ* hybridization (FISH) was occasionally used for the detection of chromosomal translocation t(14;18)(q32;q21) for the *IGH/BCL2* fusion gene, in cases where the metaphase spread was unavailable [8].

Sample collection and DNA extraction

Genomic DNA (gDNA) was extracted from tumor specimens and from matched normal peripheral blood mononuclear cells (PBMCs) using the AllPrep DNA/RNA/Protein Mini Kit (QIAGEN, Valencia, CA, USA), and the QIAamp DNA Blood Mini Kit (QIAGEN), respectively. The concentration, purity, and size of the extracted gDNA were checked using a NanoDrop 2000 spectrophotometer (Thermo Scientific, Waltham, MA, USA), Qubit 2.0 fluorometer (Thermo Fisher Scientific), and agarose gel electrophoresis.

Library preparation and whole exome sequencing (WES)

Next generation sequencing (NGS) libraries were prepared from the extracted gDNA using a SureSelect XT Target Enrichment System Kit (Agilent Technologies, Santa Clara, CA, USA) for Illumina Multiplexed Sequencing. In brief, 3.0 µg of gDNA *per* sample was fragmented into 150-200 base pairs (bps) using a Covaris S220 sonicator (Covaris, Woburn, MA, USA), and the size distribution of the products was precisely validated with a Bioanalyzer DNA 1000 (Agilent Technologies). The fragmented DNA was then end-repaired, adenylated at the 3' end, and ligated with paired-end sequencing adaptors. After amplification by the polymerase chain reaction (PCR) and removal of the unligated adaptors using Agencourt AMPure XP beads (Beckman Coulter, Tokyo, Japan), the generated libraries were assessed for their qualities and quantities using the Bioanalyzer DNA 1000. Seven hundred and fifty ng of each library was hybridized in solution with the biotinylated probes derived from the SureSelectXT Human All Exon v5 Capture Library Kit (Agilent Technologies) for 20 hours at 65°C. The hybridized fraction in the mixture was isolated by streptavidin magnetic beads, followed by a 21-cycle of PCR amplification. Each enriched library was then qualified and quantified by both the

Bioanalyzer High sensitivity assay (Agilent Technologies) and quantitative PCR using the KAPA NGS qPCR kit (Kapa Biosystems, Wilmington, MA). Finally, the sequencing was performed in the NGS Core Facility at Kyoto Prefectural University of Medicine with the HiScanSQ (Illumina, San Diego, CA, USA) in the NGS Sequencing Core in the facility using the 100-bp paired-end method for 2 runs. Pooled tumor and germline NGS libraries were loaded onto 6 lanes and 2 lanes of the flowcell for each run, respectively.

Detection of single nucleotide variants (SNVs), insertions and deletions (InDels) with the WES data

NGS raw data were converted into FASTQ files using CASAVA software (version 1.8.2), and each data set was aligned to the human genome reference (UCSC hg19) using the Burrows-Wheeler Aligner (BWA) (version 0.7.12) [9]. For quality control, we removed the low quality reads if: 1) over 80% of the sequences consisted of the bases below a quality score of 20 and 2) lengths were shorter than 30 bps. In addition, 3' end bases below a quality score of 20 were trimmed using PRINSEQ (version 0.20.4) and FASTX (version 0.0.13) [10]. Filtered and quality-controlled sequence set was aligned to the human genome reference (UCSC hg19) using the Burrows-Wheeler Aligner (BWA) (version 0.7.12) [9]. In order to summarize the basic quality of the sequencing data, we calculated the average depth of coverage as "total number of reads mapped to the reference/total number of target regions", the coverage rate of target regions as "number of bases covered over 10 reads/total bases of the targets" and the on-target rate as "total number of reads mapped to the targets/total number of reads mapped to the reference". SnpEff (version 4.0) was used to annotate SNVs and InDels [11]. PolyPhen-2 and SIFT were utilized to evaluate the possible impacts of the variants on the protein [12,13]. To investigate the functional relationships among the mutated genes, both a gene set enrichment analysis using a hypergeometric test (BiNGO) (version 3.0.3) and

pathway analysis were performed by comparing the pathway data derived from the Kyoto Encyclopedia of Genes and Genomes (KEGG) using Benjamini-Hochberg false discovery rate (FDR) correction [14]. MutSigCV (version 1.4) was used to identify driver mutation genes [15].

Supplementary References

1 Brice, P. *et al.* Comparison in low-tumor-burden follicular lymphomas between an initial no-treatment policy, prednimustine, or interferon alfa: a randomized study from the Groupe d'Etude des Lymphomes Folliculaires. Groupe d'Etude des Lymphomes de l'Adulte. *J Clin Oncol* **15**, 1110-1117 (1997).

2 Cheson, B. D. *et al.* Recommendations for initial evaluation, staging, and response assessment of Hodgkin and non-Hodgkin lymphoma: the Lugano classification. *J Clin Oncol* **32**, 3059-3068, doi:10.1200/JCO.2013.54.8800 (2014).

3 Solal-Celigny, P. *et al.* Follicular lymphoma international prognostic index. *Blood* **104**, 1258-1265, doi:10.1182/blood-2003-12-4434 (2004).

4 Federico, M. *et al.* Follicular lymphoma international prognostic index 2: a new prognostic index for follicular lymphoma developed by the international follicular lymphoma prognostic factor project. *J Clin Oncol* **27**, 4555-4562, doi:10.1200/JCO.2008.21.3991 (2009).

5 Cheson, B. D. *et al.* Report of an international workshop to standardize response criteria for non-Hodgkin's lymphomas. NCI Sponsored International Working Group. *J Clin Oncol* **17**, 1244 (1999).

6 Cheson, B. D. *et al.* Revised response criteria for malignant lymphoma. *J Clin Oncol* **25**, 579-586, doi:10.1200/JCO.2006.09.2403 (2007).

7 Shaffer, L., McGowan-Jordan, J. & Shmid, M. An International System for Human Cytogenetic Nomenclature, (S Karger, Basel 2013). *ISCN (2013)* (2013).

8 Takimoto, T. *et al.* Extranodal marginal zone lymphoma of uterine cervix with concomitant copy number gains of the MALT1 and BCL2 genes. *Oncol Lett* **in press**.

9 Li, H. & Durbin, R. Fast and accurate short read alignment with Burrows-Wheeler transform. *Bioinformatics* **25**, 1754-1760, doi:10.1093/bioinformatics/btp324 (2009).

10 Schmieder, R. & Edwards, R. Quality control and preprocessing of metagenomic datasets. *Bioinformatics* **27**, 863-864, doi:10.1093/bioinformatics/btr026 (2011).

11 Cingolani, P. *et al.* A program for annotating and predicting the effects of single nucleotide polymorphisms, SnpEff: SNPs in the genome of Drosophila melanogaster strain w1118; iso-2; iso-3. *Fly (Austin)* **6**, 80-92, doi:10.4161/fly.19695 (2012).

12 Ng, P. C. SIFT: predicting amino acid changes that affect protein function. *Nucleic Acids Research*

31, 3812-3814, doi:10.1093/nar/gkg509 (2003).

13 Adzhubei, I. A. *et al.* A method and server for predicting damaging missense mutations. *Nat Methods* **7**, 248-249, doi:10.1038/nmeth0410-248 (2010).

14 Maere, S., Heymans, K. & Kuiper, M. BiNGO: a Cytoscape plugin to assess overrepresentation of gene ontology categories in biological networks. *Bioinformatics* **21**, 3448-3449, doi:10.1093/bioinformatics/bti551 (2005).

15 Lawrence, M. S. *et al.* Mutational heterogeneity in cancer and the search for new cancer-associated genes. *Nature* **499**, 214-218, doi:10.1038/nature12213 (2013).

Supplementary Figure S1

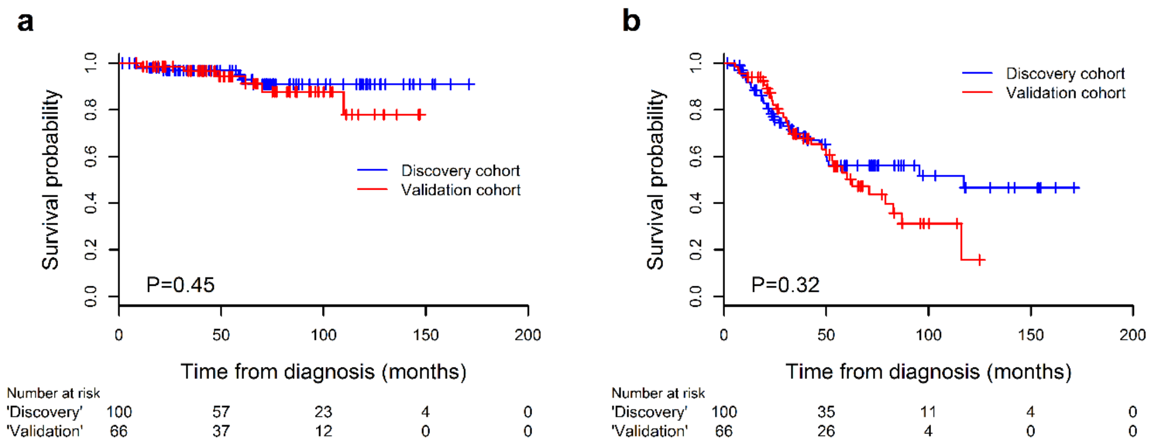

Supplementary Figure S1.

Overall survival and progression-free survival.

Overall survival (OS) (**a**) and progression-free survival (PFS) (**b**) of entire cohort. Five-year OS and PFS in the discovery cohort were 94.9% and 56.1%, respectively, and 94.3% and 53.0% in the validation cohort.

Supplementary Figure S2

a

H1	H2	H3	H4	H5
PANK4	LAPTM5	TNFRSF14	FUBP1	FUBP1
TNFRSF14	LRRC41	SFN	PPIAL4G	KIF21B
HIVEP3	GBP4	GJB4	CTSS	TAF1A
ENSA	HRNR	IGSF9	LGR6	LYST
POGZ	KPRP	SRGAP2	DHX15	OR2L8
RABGAP1L	OR6K2	ANTXR1	FDCSP	CCDC138
IRF2BP2	DDR2	NCKAP1	PTPN13	DAPL1
RYR2	DNAH14	NAT6	PCDH10	PLCD4
FMNL2	DISC1	DOCK3	HMGB2	ATP13A4
TGFBR2	HNRNPU	DNAH12	LSM11	ADAMTS3
MSL2	NDUFAF7	KLHL6	DOCK2	RASSF6
ZBBX	TRAF3IP1	RFC4	HIST1H1C	BMP2K
ATP13A4	CCDC13	HTT	ICA1	AGPAT9
GPR125	STXBP5L	SLC30A9	AASS	PIK3R1
FGB	SI	PPEF2	SARAF	POLK
NIM1K	KLHL6	TENM3	NOV	RASA1
MAML1	CLCN2	MAP1B	RAD23B	ZNF608
EEF1A1	ANK2	STK32A	LMX1B	FAM13B
CYP3A7	JADE1	FAT2	CHAT	PCDHA2
LAMB1	SPEF2	TNXB	PPP2R2D	ARAP3
CNTNAP2	MROH2B	PTCRA	NUP98	EPHA7
TNFRSF10A	SRA1	COL12A1	PGR	SOBP
EPPK1	PCDHA9	TXLNB	ZNF202	C6orf211
PLEC	CDHR2	CARD11	ETS1	PCLO
POMT1	VARS	MIOS	ANO4	CUX1
AKR1C2	PIM1	ZNF735P	SPRY2	MYOM2
PHRF1	FAM83B	LRRN3	BMP4	HMCN2
COLCA2	MYO6	EZH2	CREBBP	NSMF
TULP3	TPD52L1	FAM167A	NDRG4	PARD3
PIK3C2G	VIP	MTUS1	CA5A	CDH23
KMT2D	SPATA31D1	CSMD3	TP53	KCNMA1
PPFIA2	SVIL	ASAP1	MYO15A	PLCE1
MGAT4C	ZNF438	FREM1	CDH2	OR52N2
TMEM119	GDF2	CORO2A	DSC1	OR2AT4
RNF219	RUFY2	SIRT1	BCL2	HDAC7
MDGA2	CDH23	MUC5B	MEF2B	KMT2D
CREBBP	DNMBP	ZNF408	EXOC3L2	ALKBH1
OR1A2	OR5D16	PCF11	BPI	THSD4
HEATR6	GPR137	MMP3	IGLL5	CLEC18C
CD79B	NAALAD2	OR8B2	MYO18B	SALL3
CD79A	PIWIL4	KIAA1033	ATF4	TMPRSS9
LMTK3	POU2AF1	SPTB	ATP6AP1	NOTCH3
PRRG2	GRIN2B	SERPINA2		TRPM2
KRTAP10-4	MAP3K12	OCA2		EP300
PLA2G6	NCKAP1L	DAPK2		DCAF12L2
CHDC2	ASCL1	CREBBP		MAGEC3
CDR1	CIDEB	ERAL1		
	GSG1L	MYO1D		
	MBTPS1	C17orf104		
	DNAH2	GNA13		
	KRTAP4-7	TBCD		
	ZNF521	CNTD2		
	DTNA	SYNGR4		
	MBD1	CHD6		
	BCL2	GRIK1		
	CYP4F22	IGSF5		
	MEF2B	SUSD2		
	ZNF575	KIAA1671		
	USP29	TLR8		
	IGLL5	FANCB		
	BCR	MAGEB10		
	ARSD	TBC1D8B		
	DMD	RGAG1		
	ATP7A	SMARCA1		
	DRP2	NSDHL		

Supplementary Figure S2.
List of non-synonymous variants.
Non-synonymous variants of the high- (**a**) and standard-risk (**b**) FLs. Blue letters indicate missense mutations, mostly due to single nucleotide variations. Green, nonsense mutations; purple, frame-shift; orange, splice site mutations; red, multiple mutations.

Supplementary Figure S2 (continued)

b

S1	S2	S3	S4	S5	S6	S7	S8	S9
MST1L	PRAMEF8	TNFRSF14	KIF17	MASP2	TNFRSF14	TNFRSF14	CAPZB	NOL9
KLHDC7A	CROCC	NBPF1	ABCA4	FLG	USP24	ARID1A	LEPRE1	NBPF1
EPHA8	CYP4A22	RBMXL1	CLEC4F	PLEKHH2	SLC44A5	ZMYM4	ASB17	HS2ST1
EPHB2	CTSS	ATP1A1	ARHGEF4	MSH6	IGSF3	JTB	APOB	YY1AP1
NEXN	TTC21B	TBX15	PARP3	MAP4K4	PPIAL4G	USP21	NGEF	USH2A
BRDT	PRR21	KRTCAP3	ZBTB49	HS6ST1	NBPF10	POTEF	GOLGA4	MYO7B
CEP350	NMNAT3	STK11IP	GABRA4	ZNF804A	FLG	PHOSPHO	JARID2	MAP3K19
PRG4	INTU	ITPR1	ARHGAP10	RFTN1	KALRN	HHATL	HIST1H1D	FGD5
LGR6	PIM1	KALRN	GUCY1B3	APEH	CDV3	GNAI2	PIM1	HYAL1
NTSR2	GPR37	HGFAC	COL19A1	FAT1	ANKRD17	PRICKLE2	PCLO	CNTN3
TRIM54	DMRT3	PIK3R1	PMS2	RASGRF2	GRIA2	PIM1	DOCK4	EPHA6
FSIP2	ASCL2	ZFYVE16	SEMA3D	PCDHGB4	GEMIN5	PIM1	SYBU	MORC1
CYP8B1	OR8H3	PCDHB15	FAM86B1	ECI2	IMPG1	ZUFSP	IFNA2	ATP13A4
CLEC3B	PRB1	EBF1	DCAF4L2	PIM1	TNFAIP3	SEMA3C	FANK1	WNT8A
VPRBP	NYNRIN	TNXB	SLC28A3	SGK1	PPP1R3A	ZNF804B	KIAA1377	PCDHGB4
VPRBP	CREBBP	UFL1	ZER1	CACNA2D1	PRRC2B	MUC12	RXFP2	HIST1H4H
ARHGEF3	PKD1L3	PKD1L1	POMT1	CSMD3	ADAMTS13	ITIH2	VRK1	OR2H1
PTPRG	KRT12	KIF24	MUC2	EPPK1	PRLHR	NLRP14	NOD2	PHIP
KLHL6	RNF213	ABCA2	MMP27	COL27A1	KMT2D	LUZP2	PSG6	TULP4
WHSC1	MYL12B	AFAP1L2	HVCN1	GPR26	STAT6	NPAS3	LILRA4	EGFR
N4BP2	BCL2	MKI67	TBX3	NUMA1	LRIG3	ACTR10	SLC2A10	MAGI2
ERVMER34-1	CLEC11A	PC	FOXN3	A2ML1	P2RX4	SMOC1	ZNF831	ASNS
EPHA5	TOP3B	KMT2D	TICRR	TBC1D4	VPS33B	CREBBP	KRTAP10-2	DPP6
C4orf45	IGLL5	STAT6	HAGHL	BCL2L2	SLC5A10	SRCAP	DCAF12L1	COL14A1
AHRR	LHFPL1	USP44	CREBBP	OCA2	LRRC37A2	IL17C		TPRN
ARRDC3	UBE2A	DDHD1	C17orf112	CREBBP	10-Mar	FBXW10		ZFAND4
EBF1	SMARCA1	POTEB2	DSG2	HYDIN	BAIAP2	BCL2		REEP3
C6orf62	F9	ZNF747	PALM3	IRF8	EPG5	MUC16		CDH23
ZBED9	ATP6AP1	FAM192A	FAM71E2	SPG7	CDH20	CD22		DMBT1
IGF2R		CNTNAP1	NLRP11	BCL2	RHPN2	IGLL5		ETS1
ACTB		ASXL3	CCNB3	NOTCH3	KCNK15	FGF16		WNK1
HECW1		BCL2	USP26	RASIP1	IGLL5			GNB3
CNTNAP2		ZNF100	SLITRK4	TP53TG5	EP300			STRAP
STAU2		RHPN2		BAGE	BRD1			KMT2D
MALRD1		ZNF573		BAGE4	CNKSR2			TRHDE
RBP3		CYP2F1		BCR	SMC1A			UBC
BLNK		FLRT3		PLA2G3	CYLC1			MYO16
SH3PXD2A		PTPRT		TXLNG	IL13RA2			RYR3
MUC2		ZMYND8		TAF1				CREBBP
DPY19L2		IGLL5		RNF113A				ESRP2
CAND1		UBA1		GPR112				
FMN1		GNL3L						
CASC5		BTK						
SIN3A								
CREBBP								
PDXDC1								
IGSF6								
ITGAD								
CPNE2								
ZFHX3								
KCTD11								
EPN2								
ULK2								
QRICH2								
KCNG2								
PTPRS								

Supplementary Figure S3

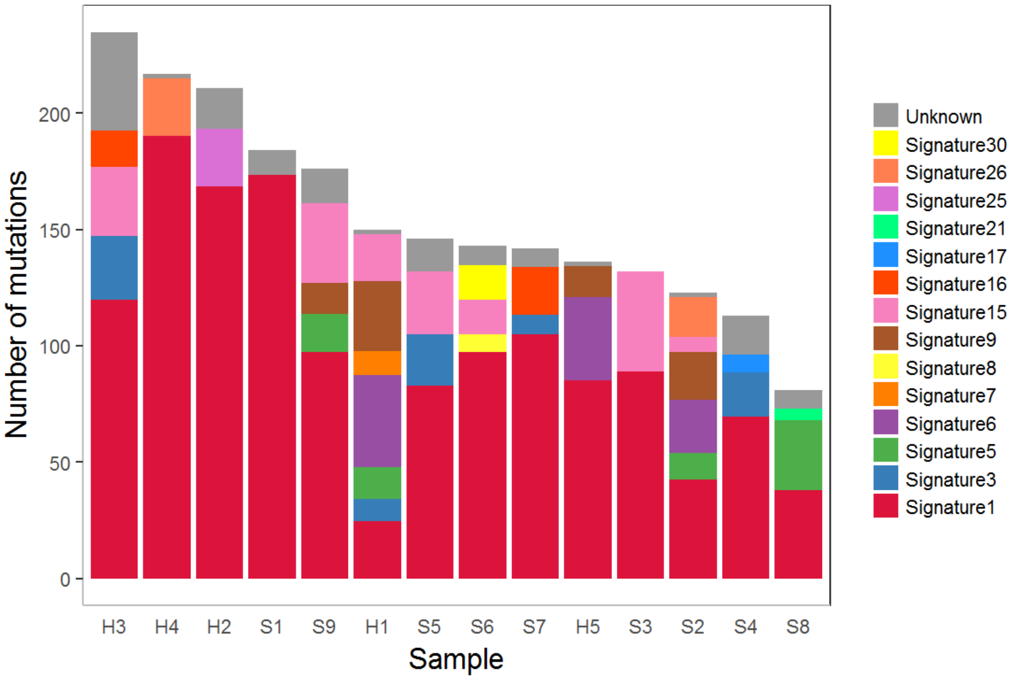

Supplementary Figure S3.
Mutational signatures of 14 FLs.
The number of mutations of each signature according to COSMIC database.

Supplementary Figure S4

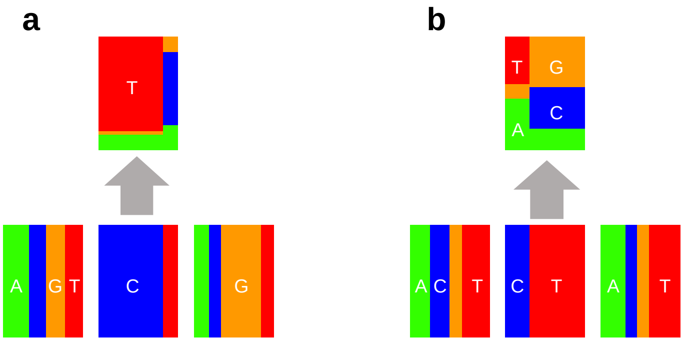

Supplementary Figure S4.
Mutational signatures of COSMIC database.
The profile of signature 1 (**a**) and signature 9 (**b**) according to COSMIC database is reconstructed into simplified logo in order to compare Figure 4a.

Supplementary Figure S5

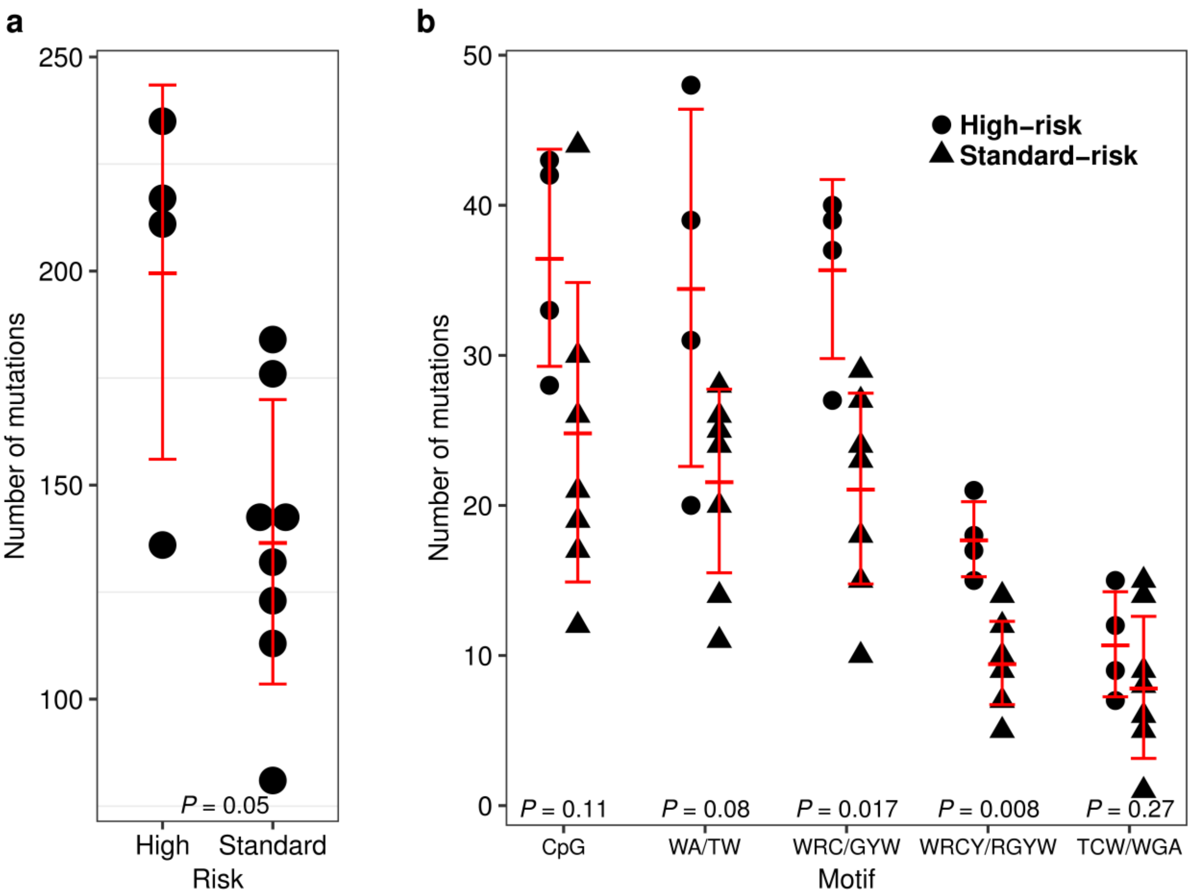

Supplementary Figure S5.

Comparison of mutational load excluding t(14;18)-negative patients.

The numbers of all somatic mutations (**a**) and mutations in CpG, WA, WRC, WRCY and TCW motifs (**b**) are indicated. Patients without t(14;18) are excluded.

Supplementary Table S1. Clinical characteristics of non-transformed follicular lymphoma (FL) patients

Characteristics	Discovery cohort (n=100)	Validation cohort (n=66)	*P*
Follow-up period, months, median (range)	61 (3-173)	55 (8-147)	0.74 ∗
Age at diagnosis, years, median (range)	61 (38-88)	63 (41-86)	0.11 ∗
Gender, male : female	42 : 58	29 : 37	0.87 †
Ann Arbor stage: 3-4	66 (66%)	46 (70%)	0.61 †
B symptom	10 (14%)	6 (13%)	1 †
Histologic grade			
1-2	75 (82%)	35 (59%)	<0.001 †
3a	10 (18%)	24 (41%)	
BM involvement	31 (31%)	24 (36%)	0.50 †
Max diameter: ≥6cm	12 (12%)	6 (9%)	1 †
No. of nodal sites: ≥5	31 (32%)	25 (46%)	0.11 †
No. of extranodal sites			
1	34 (34%)	29 (45%)	0.42 †
≥2	13 (13%)	8 (12%)	
Karyotype			
t(14;18)(q32;q21)	65 (88%)	19 (76%)	0.18 †
Normal	3 (4%)	1 (4%)	
Others	6 (8%)	5 (20%)	
FLIPI			
Low	41 (42%)	19 (32%)	0.21 †
Intermediate	27 (28%)	15 (25%)	
High	29 (30%)	26 (43%)	
FLIPI2			
Low	8 (16%)	8 (22%)	0.72 †
Intermediate	32 (63%)	20 (56%)	
High	11 (22%)	8 (22%)	

BM, bone marrow; FLIPI, follicular lymphoma international prognostic index; FLIPI2, follicular lymphoma international prognostic index-2; ∗, t-test; †, Fisher's exact test.

Supplementary Table S2. First line treatment of 103 patients in the discovery cohort

Treatment		n=100
R-CHOP/THP-COP		68 (68%)
R-monotherapy		9 (9%)
Other immunochemotherapies (R-bendamustine, R-cladribine)		7 (7%)
Watch and wait		16 (16%)
Additional treatment	R maintenance	17 (17%)
	Radiation	1 (1%)

R, rituximab.

Supplementary Table S3. Statistical analyses for extranodal involvement sites associated with disease risk in the discovery cohort

Extranodal site	Patients, N (%)	Univariate analysis		Multivariate analysis	
		HR (95% CI)	*P*	HR (95% CI)	*P*
BM	30 (30%)	1.28(0.65-2.53)	0.48		
PB	7 (7%)	4.20(1.44-12.24)	0.009	3.91(1.487-10.28)	0.006
Bone	6 (6%)	3.42(1.32-8.88)	0.012	4.90(1.65-14.5)	0.004
Liver	3 (3%)	3.30(0.79-13.82)	0.10		
Lung	1 (1%)	23.33(2.61-208.8)	0.005		
Soft tissue	4 (4%)	2.31(0.55-9.66)	0.25		
Pleura	1 (1%)	2.73(0.37-20.27)	0.33		
Duodenum	7 (7%)	0.28(0.039-2.06)	0.21		
GI excluding duodenum	6 (6%)	0.71(0.17-2.94)	0.63		

Univariate (left column) and multivariate (right column) analyses of extranodal involvement sites in progression-free survival. Multivariate analysis was adjusted for high risk of FLIPI. HR, hazard ratio; CI, confidence interval; N, number; BM, bone marrow; PB, peripheral blood; GI, gastrointestinal.

Supplementary Table S4. Clinical characteristics of the high- and standard-risk patients

	Discovery cohort			Validation cohort		
Characteristics	High-risk	Standard-risk	*P*	High-risk	Standard-risk	*P*
No. of patients	13	87		11	55	
Age, years, median (range)	67 (40-81)	60 (38-88)	0.61 ∗	62 (50-86)	64 (41-85)	0.86 ∗
Gender, male : female	4 : 9	38 : 49	0.55 †	4 : 7	25 : 30	0.74 †
Histologic grade						
1-2	10 (77%)	65 (89%)	0.36 †	7 (70%)	28(55%)	0.50 †
3a	3 (23%)	8 (11%)		3 (30%)	23 (45%)	
Ann Arbor stage : 3-4	13 (100%)	53 (61%)	0.04 †	11 (100%)	35 (64%)	0.03 †
No. of nodal sites: ≥5	8 (62%)	23 (27%)	0.02 †	4 (67%)	21 (44%)	0.40 †
Bulky mass	0 (0%)	6 (11%)	0.59 †	0 (0%)	6 (14%)	1 †
FLIPI						
Low	1 (8%)	40 (48%)	0.01 †	3 (38%)	16 (31%)	0.79 †
Intermediate	5 (38%)	22 (26%)		1 (13%)	14 (27%)	
High	7 (54%)	22 (26%)		4 (50%)	22 (42%)	
PFS, months, median	27.2	NA	<0.001 ‡	57.7	71.0	0.04 ‡

FLIPI, FL International Prognostic Index; PFS, progression-free survival; NA, not applicable; ∗, t-test; †, Fisher's exact test; ‡, log-rank test.

Supplementary Table S5. Clinical characteristics of 14 FLs subjected to whole exome sequencing (WES).

	Age	Gender	Histologic grade	CS	Extranodal sites	No. of nodal sites	Bulky mass	FLIPI/FLIPI2	karyotype
High-risk									
1	47	M	1	4A	BM, PB	10	No	Int/Int	46, XY, t(14;18)(q32;q21) [5]/49, idem, +X, +7, +12 [11]/50, idem, +X, +Y, +12, +der(18)t(14;18) [2]/46, XY [1]
2	75	F	3a	4A	BM, PB	7	No	High/NA	80, XXX, +add(1)(p11), der(1)t(1;9)(p11;q11), +3, -4, -4, +5, i(6)(p10), add(7)(q32)x2, -8, -9, +11, -12, t(14;18)(q32;q21)x2, -15, -16, -16, +19, -20, +21, -22, -22, -22, +r, +16mar [1]/46, XX [17]
3	80	F	2	4A	Bone	4	No	High/NA	NA
4	65	M	2	4B	BM, Bone, Pleura	9	No	High/High	46, XY, add(3)(q11.2), add(6)(q21), t(14;18)(q32;q21)[2]/47, XY, +3 [1]/46, XY [14]
5	62	F	1	4A	BM, PB	NA	No	Int/High	48, XX, +X, +add(5)(p11), t(14;18)(q32;q21) [1]/49, idem, +X [1]/46, XX[3]
Standard-risk									
1	71	F	2	4A	BM	2	No	Int/Int	48, XX, t(14;18)(q32;q21), +2mar [1]/46, XX [1]
2	38	F	1	3A	None	4	No	Low/NA	47, XX, +2, add(4)(p11), t(14;18)(q32;q21) [4]/46, XX [5]
3	70	F	2	4	BM	NA	No	NA/Int	46, XX, t(14;18)(q32;q21) [11]/46, XX [9]
4	51	F	1	4A	BM	1	No	Low/NA	46, XX, add(3)(q27), add(9)(p11) [1]/48, s1, +8, -10, +2mar [1]
5	51	F	2	4A	BM	2	No	Int/NA	46, XX
6	52	M	2	3A	None	5	No	Int/NA	48, XX, t(14;18)(q32;q21), +2mar [1]/46, XX [1]
7	55	F	2	3	None	NA	No	NA/NA	No metaphsase spreads, FISH(*IGH-BCL2*):Positive
8	80	M	1-2	4A	BM	9	No	Int/NA	No metaphase spreads, FISH(*IGH-BCL2*):Positive
9	71	F	2	4B	BM	NA	No	High/High	48, XX, add(8)(p11.2), t(14;18)(q32;q21), ider(18)(q10)t(14;18), +mar1

CS, clinical stage; FLIPI, follicular lymphoma international prognostic index; FLIPI2, follicular lymphoma international prognostic index-2; M, male; F, female; BM, bone marrow; PB, peripheral blood; NA, not accessed; FISH, fluorescence in situ hybridization.

Supplementary Table S6. Summary of sequencing data.

Case	Total sequenced reads	Average depth of coverage per region	Coverage rate of target regions (%)	On-target rate (%)
Tumor				
H-1	70435972	305.7	98.4	80.9
H-2	54638314	237.1	98.0	83.3
H-3	68870231	298.9	98.5	85.3
H-4	66780005	289.8	98.6	83.6
H-5	56591756	245.6	98.7	76.8
S-1	64005547	277.8	98.3	84.7
S-2	76946814	333.9	98.5	84.7
S-3	64302003	279.1	98.5	84.9
S-4	71535813	310.5	98.6	84.9
S-5	71058750	308.4	99.1	76.7
S-6	67187277	291.6	99.1	75.4
S-7	61786457	268.1	98.8	75.2
S-8	87646164	380.4	99.1	78.2
S-9	57607047	250	98.7	76.1
Germline				
H-1	23664216	102.7	91.1	82.2
H-2	26540118	115.2	92.9	82.8
H-3	22114990	96	90.1	82.3
H-4	25798580	112	92.9	85.8
H-5	21281753	92.4	91.1	76.5
S-1	18627216	80.8	86.4	77.1
S-2	23931018	103.9	91.3	86.7
S-3	23326878	101.2	91.5	86.7
S-4	25067287	108.8	91.7	86.3
S-5	29452982	98.5	92.6	77.1
S-6	18995500	82.4	88.8	76.2
S-7	23325567	101.2	91.7	76.2
S-8	24232878	105.2	92.3	78.1
S-9	20408352	88.6	89.7	77.4

Supplementary Table S8. Significantly enriched gene ontologies (GOs) within all non-synonymous mutated genes.

GO ID	GO name	*P*	FDR
30154	cell differentiation	9.96E-08	2.86E-04
48869	cellular developmental process	3.28E-07	4.71E-04
48699	generation of neurons	6.41E-07	6.13E-04
48468	cell development	1.16E-06	8.33E-04
30182	neuron differentiation	1.96E-06	1.12E-03
32502	developmental process	2.37E-06	1.12E-03
7399	nervous system development	2.88E-06	1.12E-03
48666	neuron development	3.24E-06	1.12E-03
22008	neurogenesis	3.52E-06	1.12E-03
7275	multicellular organismal development	4.86E-06	1.24E-03
32501	multicellular organismal process	4.98E-06	1.24E-03
7417	central nervous system development	5.20E-06	1.24E-03
48731	system development	6.62E-06	1.46E-03
51153	regulation of striated muscle cell differentiation	8.85E-06	1.81E-03
48856	anatomical structure development	1.07E-05	2.05E-03
48742	regulation of skeletal muscle fiber development	1.58E-05	2.83E-03
31175	neuron projection development	3.00E-05	5.07E-03
42981	regulation of apoptosis	3.45E-05	5.50E-03
43067	regulation of programmed cell death	4.34E-05	6.40E-03
45662	negative regulation of myoblast differentiation	4.46E-05	6.40E-03
7156	homophilic cell adhesion	4.82E-05	6.41E-03
1763	morphogenesis of a branching structure	4.91E-05	6.41E-03
10941	regulation of cell death	5.28E-05	6.58E-03
23033	signaling pathway	6.33E-05	7.42E-03
23052	signaling	6.46E-05	7.42E-03
48513	organ development	7.38E-05	8.15E-03
48641	regulation of skeletal muscle tissue development	8.10E-05	8.58E-03
16043	cellular component organization	8.38E-05	8.58E-03
7155	cell adhesion	9.78E-05	9.63E-03
22610	biological adhesion	1.01E-04	9.63E-03
51147	regulation of muscle cell differentiation	1.14E-04	1.04E-02
16337	cell-cell adhesion	1.16E-04	1.04E-02

18205	peptidyl-lysine modification	1.26E-04	1.10E-02
60284	regulation of cell development	1.30E-04	1.10E-02
61138	morphogenesis of a branching epithelium	1.50E-04	1.22E-02
48522	positive regulation of cellular process	1.53E-04	1.22E-02
9792	embryonic development ending in birth or egg hatching	1.64E-04	1.27E-02
7420	brain development	2.03E-04	1.53E-02
51128	regulation of cellular component organization	2.25E-04	1.66E-02
2009	morphogenesis of an epithelium	3.42E-04	2.45E-02
43009	chordate embryonic development	3.51E-04	2.46E-02
48518	positive regulation of biological process	3.75E-04	2.56E-02
7584	response to nutrient	3.91E-04	2.61E-02
45661	regulation of myoblast differentiation	4.06E-04	2.65E-02
22612	gland morphogenesis	4.57E-04	2.86E-02
7569	cell aging	4.58E-04	2.86E-02
46578	regulation of Ras protein signal transduction	4.90E-04	2.99E-02
10038	response to metal ion	5.19E-04	3.11E-02
16202	regulation of striated muscle tissue development	5.60E-04	3.28E-02
1701	in utero embryonic development	5.77E-04	3.31E-02
48634	regulation of muscle organ development	6.34E-04	3.56E-02
48812	neuron projection morphogenesis	7.09E-04	3.88E-02
45595	regulation of cell differentiation	7.25E-04	3.88E-02
51148	negative regulation of muscle cell differentiation	7.40E-04	3.88E-02
31667	response to nutrient levels	7.51E-04	3.88E-02
33189	response to vitamin A	7.58E-04	3.88E-02
51095	regulation of helicase activity	7.79E-04	3.92E-02
902	cell morphogenesis	8.39E-04	4.09E-02
51097	negative regulation of helicase activity	8.40E-04	4.09E-02
35023	regulation of Rho protein signal transduction	9.42E-04	4.34E-02
30030	cell projection organization	9.46E-04	4.34E-02
1101	response to acid	9.57E-04	4.34E-02
30199	collagen fibril organization	9.57E-04	4.34E-02
51056	regulation of small GTPase mediated signal transduction	9.70E-04	4.34E-02
51960	regulation of nervous system development	9.83E-04	4.34E-02
35295	tube development	1.11E-03	4.80E-02
7431	salivary gland development	1.14E-03	4.80E-02

2520	immune system development	1.15E-03	4.80E-02
50767	regulation of neurogenesis	1.17E-03	4.80E-02
43065	positive regulation of apoptosis	1.17E-03	4.80E-02
50794	regulation of cellular process	1.22E-03	4.93E-02

FDR, false discovery rate.

Supplementary Table S9. Significantly enriched Kyoto Encyclopedia of Genes and Genome (KEGG) pathways within all non-synonymous mutated genes.

KEGG_ID	Pathway name	*P*	FDR
hsa04919	Thyroid hormone signaling pathway	0.00075	0.197814
hsa04360	Axon guidance	0.0036	0.197814
hsa05206	MicroRNAs in cancer	0.00441	0.197814
hsa04320	Dorso-ventral axis formation	0.00967	0.235867
hsa04970	Salivary secretion	0.0103	0.235867
hsa04662	B cell receptor signaling pathway	0.01038	0.235867
hsa04520	Adherens junction	0.01107	0.235867
hsa04611	Platelet activation	0.0116	0.235867
hsa04713	Circadian entrainment	0.01436	0.273738
hsa04330	Notch signaling pathway	0.02209	0.313752
hsa04720	Long-term potentiation	0.02366	0.313752
hsa05215	Prostate cancer	0.02734	0.347446
hsa03010	Ribosome	0.0342	0.355325
hsa05412	Arrhythmogenic right ventricular cardiomyopathy (ARVC)	0.03495	0.355325
hsa04921	Oxytocin signaling pathway	0.03878	0.369622
hsa04960	Aldosterone-regulated sodium reabsorption	0.04071	0.376259
hsa03430	Mismatch repair	0.04286	0.384479
hsa04730	Long-term depression	0.04728	0.4120114
